# DArTseq-Based High-Throughput SilicoDArT and SNP Markers Applied for Association Mapping of Genes Related to Maize Morphology

**DOI:** 10.3390/ijms22115840

**Published:** 2021-05-29

**Authors:** Agnieszka Tomkowiak, Jan Bocianowski, Julia Spychała, Joanna Grynia, Aleksandra Sobiech, Przemysław Łukasz Kowalczewski

**Affiliations:** 1 Department of Genetics and Plant Breeding, Poznań University of Life Sciences, 11 Dojazd St., 60-632 Poznań, Poland; julia.spychala96@gmail.com (J.S.); a.sobiech1404@gmail.com (A.S.); 2 Department of Mathematical and Statistical Methods, Poznań University of Life Sciences, 28 Wojska Polskiego St., 60-637 Poznań, Poland; jan.bocianowski@up.poznan.pl; 3 Poznań Plant Breeding Sp. z o. o., 5 Kasztanowa St., 63-004 Tulce, Poland; joannagrynia@wp.pl; 4 Department of Food Technology of Plant Origin, Poznań University of Life Sciences, 31 Wojska Polskiego St., 60-624 Poznań, Poland; przemyslaw.kowalczewski@up.poznan.pl

**Keywords:** DArTSeq, SNP, candidate gene association mapping, genome-wide association mapping, morphological features, *Zea mays* L.

## Abstract

Today, agricultural productivity is essential to meet the needs of a growing population, and is also a key tool in coping with climate change. Innovative plant breeding technologies such as molecular markers, phenotyping, genotyping, the CRISPR/Cas method and next-generation sequencing can help agriculture meet the challenges of the 21st century more effectively. Therefore, the aim of the research was to identify single-nucleotide polymorphisms (SNPs) and SilicoDArT markers related to select morphological features determining the yield in maize. The plant material consisted of ninety-four inbred lines of maize of various origins. These lines were phenotyped under field conditions. A total of 14 morphological features was analyzed. The DArTseq method was chosen for genotyping because this technique reduces the complexity of the genome by restriction enzyme digestion. Subsequently, short fragment sequencing was used. The choice of a combination of restrictases allowed the isolation of highly informative low copy fragments of the genome. Thanks to this method, 90% of the obtained DArTseq markers are complementary to the unique sequences of the genome. All the observed features were normally distributed. Analysis of variance indicated that the main effect of lines was statistically significant (*p* < 0.001) for all 14 traits of study. Thanks to the DArTseq analysis with the use of next-generation sequencing (NGS) in the studied plant material, it was possible to identify 49,911 polymorphisms, of which 33,452 are SilicoDArT markers and the remaining 16,459 are SNP markers. Among those mentioned, two markers associated with four analyzed traits deserved special attention: SNP (4578734) and SilicoDArT (4778900). SNP marker 4578734 was associated with the following features: anthocyanin coloration of cob glumes, number of days from sowing to anthesis, number of days from sowing to silk emergence and anthocyanin coloration of internodes. SilicoDArT marker 4778900 was associated with the following features: number of days from sowing to anthesis, number of days from sowing to silk emergence, tassel: angle between the axis and lateral branches and plant height. Sequences with a length of 71 bp were used for physical mapping. The BLAST and EnsemblPlants databases were searched against the maize genome to identify the positions of both markers. Marker 4578734 was localized on chromosome 7, the closest gene was Zm00001d022467, approximately 55 Kb apart, encoding anthocyanidin 3-*O*-glucosyltransferase. Marker 4778900 was located on chromosome 7, at a distance of 45 Kb from the gene Zm00001d045261 encoding starch synthase I. The latter observation indicated that these flanking SilicoDArT and SNP markers were not in a state of linkage disequilibrium.

## 1. Introduction

A stronger emphasis on increased and more balanced nutritional production has been noticeable for a long time. Therefore, newer tools are continuously being developed to guarantee a better accuracy of selection, including maize [1]. The currently used selection methods have been complemented with molecular biology findings and statistical models enabling both the identification of markers of individual traits, resulting from the action of individual genes, and those conditioned by many QTLs that explain the phenotypic variability of a trait to a varying degree [2,3]. The relationship between phenotypic and genetic variability can be analyzed using association mapping, also called linkage disequilibrium [4,5,6].

We can distinguish two approaches in association mapping. The first one is candidate gene association and the second is genome-wide association study (GWAS). As regards candidate gene association, the following hypothesis is tested: “Is there a correlation between a DNA polymorphism in a specific gene and a trait?” GWAS approach is justified in the absence of detailed biochemical knowledge related to the sought trait. GWAS searches for a genome-wide trait-marker association and assumes that there are markers exhibiting linkage disequilibrium in the genome conditioning trait expression [7]. The association mapping was first performed in maize in 1995 [8]. Next-generation sequencing (NGS) is applied to identify SNP and SilicoDArT markers.

Currently, the DArT platform offers analyses based on the NGS-DArTseq technology [9]. The DArTseq method reduces the complexity of the genome by digestion with restriction enzymes followed by sequencing of short reads. The choice of a combination of restriction enzymes allows for the isolation of highly informative low copy fragments of the genome. Even 90% of the obtained DArTseq markers are complementary to the unique sequences of the genome [10,11]. DArTseq analysis generates two datasets. The first one contains dominant markers; the second one includes codominant markers with marked single nucleotide polymorphisms. At least three times as many dominant markers are obtained in comparison to the conventional DArT method [12]. Maize, similar to barley and rice, is one of the best-known cereal species in terms of genetics. High polymorphism is a characteristic feature of the maize genome. Several active alleles are present at many loci, and the frequency of duplicate DNA sequences, whose significant fraction are retrotransposons and transposons, is about 58%. The genic regions account for only 7.5% of the entire maize genome [13]. From the breeding perspective, genes determining the important morphological features that influence the yield are very important.

In maize breeding, great attention is paid to the selection of traits that can help achieve maximum yield. The recent increase in the level of maize yield is largely the result of the introduction of new, more fertile cultivars into practice. The potential grain yield of a given cultivar depends on various features that form the plant habit. The following morphological features are of particular importance: plant height, height of the first cob, number of days from sowing to anthesis, number of days from sowing to silk emergence, angle between the axis and lateral branches (tassel), curvature of lateral branches (tassel), length of the main axis above the highest lateral branch (tassel), number of primary lateral branches (tassel) and anthocyanin discoloration. Certain morphological features may significantly affect quantitative traits [14,15]. Intercultivar diversification of maize morphological traits may generate an unequal response of cultivars to the main agriculture practice factors (fertilization, sowing date and density) [16,17,18,19].

The results of the research prove that the height of maize plants and the height of the first cob are highly heritable features (usually > 0.70). They are conditioned by additive genes and/or genes showing overdominance or partial dominance [20]. These genes are most frequently located in the regions of chromosomes 1, 3, 7L, 8L, 9S and 10L. The authors reported that the location of some QTL regions determining maize height overlapped with the location of certain quantitative genes [21].

The study of Bódi et al. [14] demonstrated that the morphological structure of the tassel was related to the quantity of pollen produced, thus affecting the yield. Mickelson et al. [22] found that the length and weight of the tassel was negatively correlated with cob length. Growers should select cultivars with shorter and lighter tassels because this structure of male generative organs increases the yield [23]. Hallauer et al. [24] argued that a large and highly branched tassel negatively affected grain production because it competed with the cob for nutrients.

Anthocyanin discoloration is another morphological feature reflected in maize yield. The biosynthesis of flavonoid pigments is a very complex process. For example, plants discolored with anthocyanin heat up more at lower ambient temperatures and grow faster than plants without discoloration. Research has shown that the regulation of anthocyanin biosynthesis requires genes from two groups of factors that control the transcription of structural genes. These factors (their protein products) possess characteristic domains—fragments that bind to DNA at specific sites [25,26].

Therefore, the aim of the study was to identify single-nucleotide polymorphisms (SNPs) and SilicoDArT markers related to selected morphological features determining the yield in maize. These markers will be used to select parent components for heterotic crosses. The linkage disequilibrium (LD) decay was assessed to estimate the required marker number and potential resolution in GWAS.

## 2. Results

### 2.1. Phenotyping

**Figure 1:**
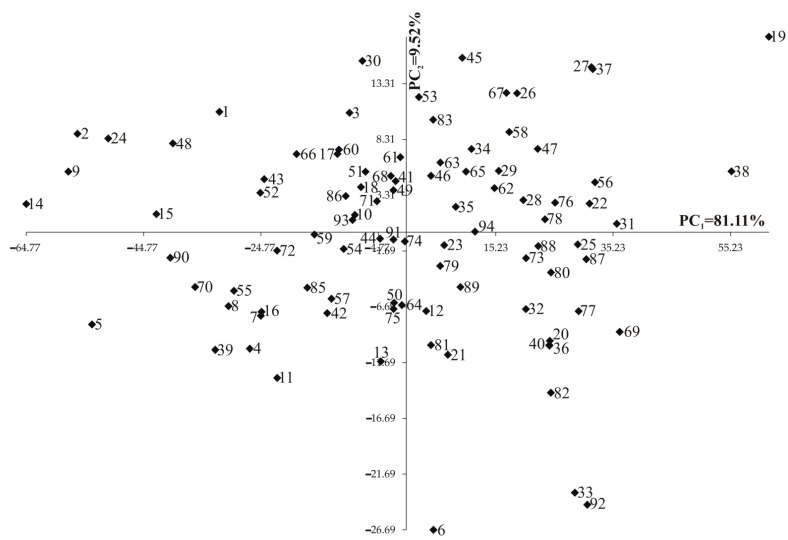
Population structure of inbred lines of maize (*Zea mays* L.) estimated by eigenanalysis. Dent ID: 5,7,8,9,20,22,23,33,43,44,56,58,59,61,62,64,65,72,76,79,80,83,85,88,89,90,94; Dent BSSS: 47,49,50,87; Dent ID/BSSS: 1,3,4,10,13,14,17,19,45,46,54,57,63,66,67,75,77,78,81,84,86,91,92; Dent Lancaster: 55; Dent ID/Lancaster: 50; Flint F2: 74; Flint F2/EP1: 2,6,15,18,21,24,35,39,41,51,53; Flint F2/CM7: 73; Flint/BSSS: 38; Flint/ID: 40; Flint/Lancaster: 16,60,68; German Flint/F2: 11,69,82,93; Flint—Origin unknown: 12,42,52,36; Semident BSSS: 30,31,32,71; Semident—Origin unknown: 25,26,27,28,29,37,34,48.

All the observed features had normal distribution. Analysis of variance indicated that the main effect of lines was statistically significant (*p* < 0.001) for all 14 traits. The values for the first two principal components were also significant and accounted jointly for 90.63% of the whole variation (Figure 1). The tested lines did not cluster according to the type of the grain. The generated groups included both flint and dent grain lines (Figure 1).

**Figure 2:**
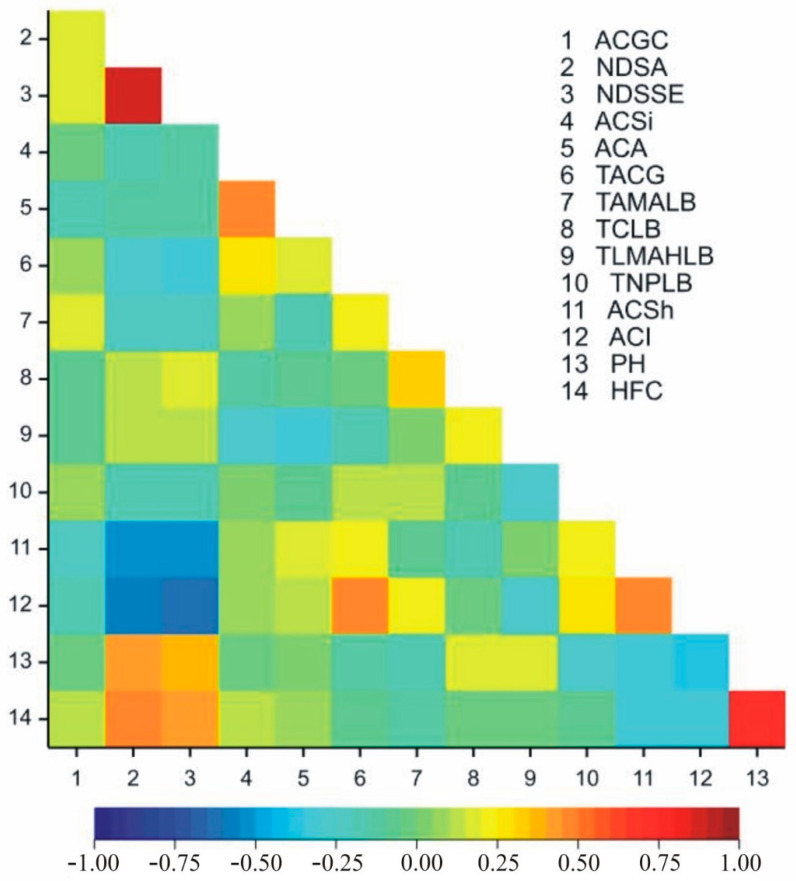
Heatmaps for linear Pearson’s correlation coefficients between the observed traits on the basis of mean values for inbred lines. ACGC-anthocyanin coloration of cob glumes, NDSA—number of days from sowing to anthesis, NDSSE—number of days from sowing to silk emergence, ACSi-anthocyanin coloration of silks, ACA-anthocyanin coloration of anthers, TACG-tassel: anthocyanin coloration at glume base, TAMALB—tassel: angle between the axis and lateral branches, TCLB—tassel: curvature of lateral branches, TLMAHLB—tassell: length of the main axis above the highest lateral branch, TNPLB—tassel: number of primary lateral branches, ACSh—anthocyanin coloration of the sheath, ACI—anthocyanin coloration of internodes, PH—plant height, HFC—height of the first cob.

Figure 2 shows correlation coefficients between the observed traits. A positive correlation was observed between the following trait pairs: NDSA-DNSSE (*r* = 0.864), NDSA-PH (0.440), NDSA-HFC (0.494), NDSSE-PH (0.384),NDSSE-HFC (0.425), ACSi-ACA (0.451), ACSi-ACA (0.287), TACG-ACSh (0.247), TACG-ACI (0.461), TAMALB-TCLB (0.305), TAMALB-ACI (0.234), TCLB-TLMAHLB (0.227), TNPLB-ACSh (0.223), TNPLB-ACI (0.277), ACSh-ACI (0.489), and PH-HFC (0.722) (Figure 2). The negative correlation was observed between the following trait pairs: ACGC-ACSh (−0.237), NDSA-TACG (−0.264), NDSA-TAMALB (−0.242), NDSA-ACSh (−0.530), NDSA-ACI (−0.591), NDSSE-TACG (−0.325), NDSSE-TAMALB (−0.225), NDSSE-ACSh (−0.548), NDSSE-ACI (−0.617), ACSi-TLMAHLB (−0.269), ACA-TLMAHLB (−0.315), TLMAHLB-TNPLB (−0.252), TLMAHLB-ACI (−0.254), TNPLB-PH (−0.254), ACSh-PH (−0.330), ACSh-HFC (−0.341), ACI-PH (−0.399), and ACI-HFC (−0.307) (Figure 2).

### 2.2. Genotyping Data (SilicoDArT and SNP)

**Figure 3:**
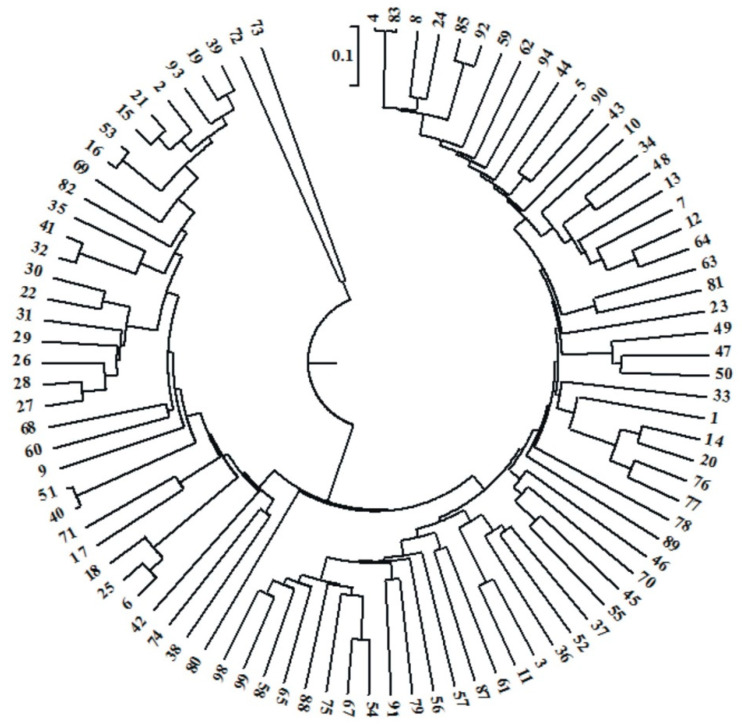
Dendrogram of genetic similarity of the studied inbred lines of maize (*Zea mays* L.) on the basis of single-nucleotide polymorphism (SNP) and SilicoDArT marker observations.

Thanks to the DArTseq analysis using next-generation sequencing (NGS) in the studied plant material, it was possible to identify 49,911 polymorphisms, of which 33,452 were SilicoDArT markers and the remaining 16,459 were SNP markers. Out of these, 6862 markers (including 5472 SilicoDArTs and 1390 SNPs) were selected for GWAM using the following criteria: one SilicoDArT and SNP within a given sequence (69 bp), minor allele frequency (MAF) >0.25 and the missing observation fractions <10%. This set of 33,452 SNP and SilicoDArT markers was utilized for the analyses of genetic variation, LD, and GWAS. Pairwise *r*^2^ values between markers were calculated to assess the overall extent of LD. The linkage disequilibrium (measured as *r*^2^) was not larger than 0.2. The unweighted pair group method with arithmetic average (UPGMA) was used for clustering. A dendrogram was plotted based on the identified SilicoDArT and SNP markers, showing the genetic similarity between the 94 inbred lines (Figure 3, Table S1).

The identified SNP and SilicoDArT markers were used to group the studied lines according to their origin. Five main similarity groups were distinguished (Table S2). The first group consisted of 33 lines. Flint lines (19 lines) were dominant in this group: there were five lines with a dent kernel type, and the remaining nine were semident lines. The highest similarity was found between the following flint lines: line 40 (Flint/ID) and line 51 (F2/EP1) were 97 percent similar, while lines 16 (Flint/Lancaster) and 53 (F2/EP1) were 95 percent similar. An equally high coefficient of similarity was recorded between lines 32 (semident BSSS) and 41 (F2/EP1)—93% (Figure 3). The second group included only one line 80 (LD), which differed from all the others by 60 percent. The third group consisted of 19 lines. It was dominated by dent grain lines (15 lines). This group also included three flint grain lines and one semident line. The highest similarity was found between the dent lines: line 54 (Flint/BSSS) and line 67 (ID/BSSS) were 81 percent similar, while lines 54 (ID/BSSS) and 67 (ID/BSSS) were 73 percent similar. Overall, Group 1, which contained the majority of flint lines, differed from Group 3, which included 62 percent of dent lines. Thus, it could be seen that SNP and SilicoDArT markers clearly differentiated the analyzed lines in terms of their origin (Figure 3). The fourth, largest group consisted of 39 inbred lines, of which the most numerous were dent grain lines (34 lines), followed by flint (three lines) and semident lines (two lines). The greatest similarity was recorded between dent grain type lines. Line No. 4 (ID/BSSS) and No. 83 (ID/BSSS) were 98 percent similar and line 85 (ID) and 92 (ID/BSSS) were 93 percent similar. The fifth and final group included two inbred lines72 (ID) and 73 (F2/CM7) (Table S2). These lines differed from each other by 84 percent and from all other lines by 62 percent. Both lines could be used for heterotic crosses.

**Figure 4:**
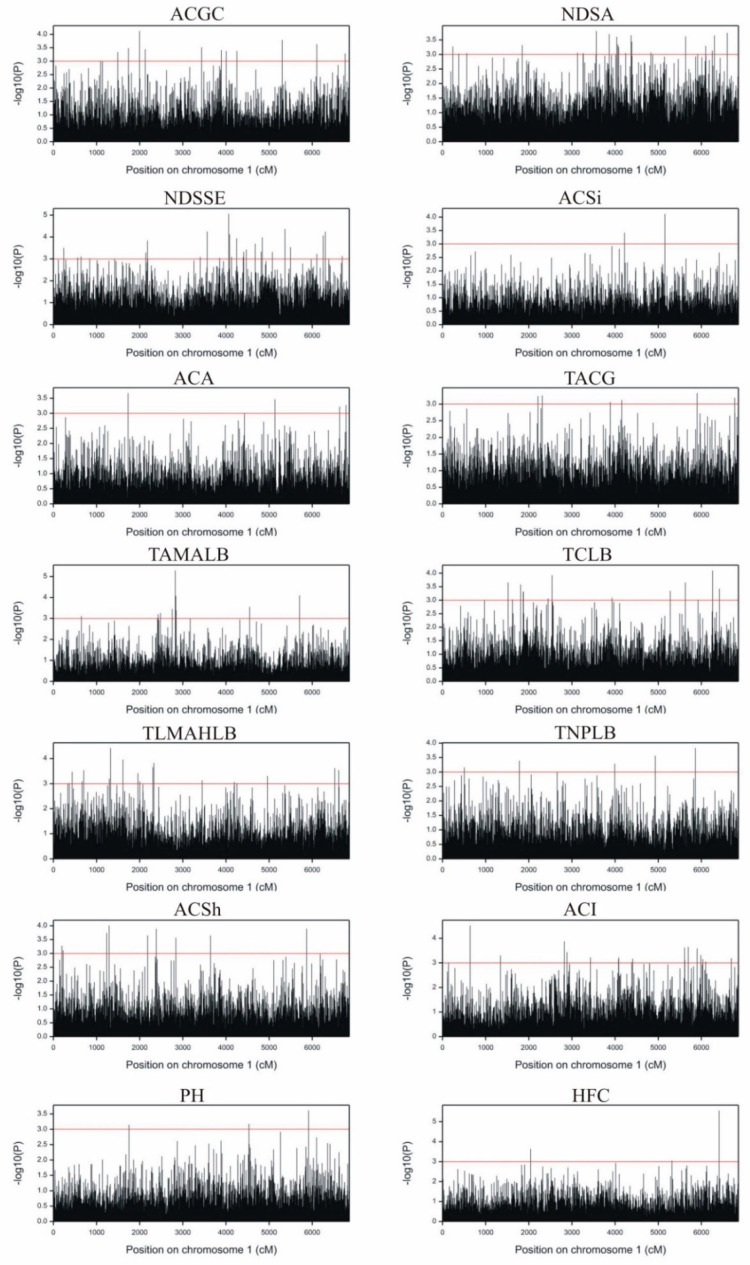
Manhattan plots for all 14 observed traits of maize: ACGC-anthocyanin coloration of cob glumes, NDSA—number of days from sowing to anthesis, NDSSE—number of days from sowing to silk emergence, ACSi-anthocyanin coloration of silks, ACA-anthocyanin coloration of anthers, TACG-tassel: anthocyanin coloration at glume base, TAMALB—tassel: angle between the axis and lateral branches, TCLB—tassel: curvature of lateral branches, TLMAHLB—tassell: length of the main axis above the highest lateral branch, TNPLB—tassel: number of primary lateral branches, ACSh—anthocyanin coloration of the sheath, ACI—anthocyanin coloration of internodes, PH—plant height, HFC—height of the first cob.

The number of markers significantly associated with the investigated traits at the false discovery rate (FDR) <0.05 was 481 in GWAM: 377 of SilicoDArTs and 104 of SNPs (Table 1). The fewest markers (10) were associated with anthocyanin coloration of silks (ACSi), and the most (77) with the number of days from sowing to time of silk emergence (NDSSE) (Table S1, Figure 4). Table 2 shows markers associated with the analyzed features. Overall, 297 SilicoDArT markers were associated with a single trait, 33 SilicoDArT markers were associated with three traits and one SilicoDArT marker was associated with four traits were identified. In the case of SNP markers, 75 of them were associated with one trait, 11 SNPs were associated with two traits, one SNP was associated with three traits, and one SNP was associated with four traits (Figure 4). Markers associated with more than one trait are characterized by pleiotropy, a trait desirable in plant breeding. Among those mentioned, two markers associated with four analyzed traits deserved special attention: SNP (4578734) and SilicoDArT (4778900) (Figure 4). SNP marker 4578734 was associated with the following features: ACGC, NDSA, NDSSE and ACI. SilicoDArT marker 4778900 was associated with the following features: NDSA, NDSSE, TAMALB and PH (Table S1). Sequences with a length of 71 bp were used for physical mapping. The BLAST and EnsemblPlants databases were searched against the maize genome to identify the positions of both markers. Marker 4578734 was localized on chromosome 7; the closest gene was Zm00001d022467, approximately 55 Kb apart, encoding anthocyanidin 3-*O*-glucosyltransferase. Marker 4778900 was localized on chromosome 9 in the vicinity (approx. 45 Kb) of the Zm00001d045261 gene, encoding starch synthase I. The latter observation indicated that these flanking SilicoDArT and SNP markers were not in a state of linkage disequilibrium.

## 3. Discussion

Technologies are increasingly used in plant breeding that allow for faster genotyping using next-generation sequencing (NGS) [27]. Modern NGS techniques are characterized by higher throughput and efficiency compared to the previously used Sanger sequencing technique [28]. Solex (Ilumina) is one of the most widely used NGS techniques. Whole-genome sequence reads, thanks to the use of NGS techniques, allow for the identification of markers associated with phenotypic changes and their further use in genomic selection (GS) or association mapping (AM) [29].

Modern genotyping methods based on next-generation sequencing (NGS) include, among others, genotyping by sequencing (GBS) [30] and DArTseq technology [31,32,33]. The DArT technology based on a hybridization technique were used to scan cereal genomes prior to the introduction of NGS-based methods [12]. The DArTseq technology is a modification of the DArT method. The hybridization step on microarrays in DArTseq technology was replaced by next-generation sequencing in the Illuminy system. Several times, more polymorphic markers—both dominant silicoDArT and codominant SNPs—are obtained as a result of these analyses.

In the present study, 49,911 polymorphisms, among which 33,452 were SilicoDArT markers and 16,459 were SNP markers, were identified as a result of next-generation sequencing. Association with selected morphological features was tested using 5472 SilicoDArT markers and 1390 SNP markers, of which 377 SilicoDArT and 104 SNP markers were statistically significantly associated with at least one observed trait. Particularly noteworthy were SNP marker 4578734 and SilicoDArT marker 4778900, which were associated with four analyzed traits (Table S1). The identified markers allowed them to estimate the genetic similarity between the studied inbred lines and to divide them according to their origin. The greatest genetic distance (84%) was found between lines 72 (ID) and 73 (F2/CM7). Both lines can be used for heterotic crosses.

DArT marker polymorphisms result from the changes identified in the regions recognized by restriction enzymes. As regards DArTseq and GBS markers, sequential changes play a more important role. The advantage of sequence markers is information about a specific sequence in the genome. This facilitates their conversion to specific markers, targeted directly at the polymorphic site [12,32,33].

In association mapping, genes that determine morphological traits can be identified through candidate gene association mapping and genome-wide association study (GWAS) [34,35]. Genome-wide association study (GWAS) has been widely applied by many authors [29,36,37]. Association mapping in maize was used to identify markers associated with important agronomic traits, such as grain yield and yield structure traits [38]. Markers linked to features associated with plant morphology also play a very important role.

The current study found that 11 markers were significantly associated with plant height (SilicoDArT and SNP jointly), and 19 markers were significantly associated with the height of the first cob. The plant flowering period (earlier forms are usually lower) and the number of internodes formed by plants during the entire growing season are essential for genotypic variability in plant height [39]. The polygenic nature of the height of maize plants and the height of the first cob was confirmed by the study of Flint-Garcia et al. [21]. The authors argued that the QTL regions responsible for plant height were located on chromosomes 1, 3, 7L, 8L, 9S and 10L, and that the QTLs conditioning height overlapped with the position of “qualitative” genes with a simple mode of inheritance. The grain yield is affected not only by plant height, but also by tassel structure. Dell’Acqua et al. [40] generated, for the first time, a balanced multi-parental population in maize, which serves as a tool for effortless QTL mapping in maize due to a large variety and dense recombination events. Authors described three flowering time QTLs and three grain yield QTLs and indicated potential candidate genes. MAGIC maize subsets have been demonstrated to acquire high power and high-resolution QTL mapping in power simulations.

Morphological tassel traits are of importance in maize breeding programs, in which inbred lines are developed with the aim of reducing the size and number of branches, while maintaining satisfactory pollen production [41,42]. The present experiments identified a total of 143 markers (SilicoDArTs and SNPs) associated with tassel morphological structure (angle between the main axis and lateral branches—27 markers, curvature of lateral branch—51 markers, main axis length above the highest lateral branch—51 markers, number of primary lateral branches—29 markers). One of these markers, which was statistically significantly associated with, *inter alia*, angle between the main axis and lateral branches was physically mapped. This marker has been localized near the Zm00001d045261 gene coding for starch synthase I, which is involved in starch synthesis. Starch synthesis is an elaborate process employing several isoforms of starch synthases (SSs), starch branching enzymes (SBEs) and debranching enzymes (DBEs). In cereals, some starch biosynthetic enzymes can form heteromeric complexes, whose assembly is controlled by protein phosphorylation [43]. Other authors [44] have reported that BAD1 is a TCP class II gene that functions to promote cell proliferation in a lateral organ, the pulvinus, and affects the inflorescence architecture by influencing lateral branch emergence angle. According to Qin et al. [45], tassel branch number (TBN) is an important agronomic trait that directly contributes to grain yield in maize (*Zea mays* L.), and the identification of genes precisely regulating TBN in the parental lines was important for maize hybrid breeding. The authors concluded that the SCF (Skp1/Cul1/F-box protein/Roc1) complex and the ABA signaling pathway might be involved in TBN modulation in maize. Anthocyanin discoloration is another important morphological feature associated with grain yield.

Anthocyanin discoloration is stimulated, i.a., by stress factors such as low temperatures, nutrient deficiency or drought. Anthocyanin biosynthesis is also dependent on light intensity; a large amount of light energy and light with blue and UV spectrum stimulate the synthesis of anthocyanins. In such cases, they play a protective role in the PSII photosynthetic system against photoinhibition [46]. Plants discolored with anthocyanin heat up more at lower ambient temperatures and grow faster than plants without discoloration. The anthocyanin, flobafen and other flavonoid biosynthetic pathways in maize tissues are controlled by more than 20 structural and regulatory genes [47]. Therefore, the search for markers linked to these genes is necessary for their rapid identification using molecular markers. The present study identified a total of 165 markers (SilicoDArTs and SNPs) associated significantly with anthocyanin coloration of individual maize plant parts (anthocyanin coloration of cob glumes—31 markers, anthocyanin coloration of silks—10 markers, anthocyanin coloration of anthers—17 markers, tassel: anthocyanin coloration at glume base—22 markers, anthocyanin coloration of the sheath—31 markers, anthocyanin coloration of internode—54 markers). One of these markers, SNP 4578734, apart from ACI, was significantly associated with three other traits (ACGC, NDSA, NDSSE). Marker 4578734 was localized on chromosome 7, near the Zm00001d022467 gene. This gene encodes anthocyanidin 3-*O*-glucosyltransferase. According to Grotewold [25], the *Bz1* gene is located on chromosome 10L, which also encodes 3GT transferase (3-*O*-glucosyltransferase), involved in the conversion of hydroxyanthocyanidine into cyanidin-3-glucoside. The dominant *Bz1* allele determines purple color of grain aleurone layer, while aleurone layers are light brown or red-brown in the presence of the recessive *bz1* allele [48].

## 4. Materials and Methods

### 4.1. Plant Material

The plant material consisted of 94 inbred maize lines. Part of the analyzed lines were characterized by dent-type kernels; they were derived from various groups of origin from the United States: Iowa Stiff Stalk Synthetic (BSSS), Iowa Dent (ID) and Lancaster. The second part of the plant material was flint grain lines of three different origins: F2 (a group related to the F2 line bred at INRA in France from the Lacaune population), EP1 (a group related to the EP1 line, bred in Spain from the population derived from the Pyrenees) and German Flint. The plant material was obtained from Polish breeding companies: Hodowla Roślin Smolice Group IHAR (Smolice, Poland) and Małopolska Hodowla Roślin (Kobierzyce, Poland). Inbred lines of complex origin bred from different starting populations and lines of unknown origin were also used in the study (Table 2).

### 4.2. Phenotyping

A field experiment with 94 inbred lines was established in 2017 on plots belonging to the Polish breeding company Hodowla Roślin Smolice Grupa IHAR sp. z o.o. (Smolice, Poland) (51°42′020.813″ N, 17°9′057.405″ E). The experiment was set up in a randomized block design, in three replicates on plots of 10 m^2^. The analysis of morphological features was conducted from May to October 2017 and included the following features: plant height (PH), height of the position of the first cob (HFC), number of days from sowing to anthesis (NDSA), number of days from sowing to silk emergence (NDSSE), anthocyanin coloration of cob glumes (ACGC), anthocyanin coloration of silks (ACSi), anthocyanin coloration of anthers (ACA), anthocyanin coloration of the sheath (ACSh), anthocyanin coloration of internodes (ACI), tassel: anthocyanin coloration at glume base (TACG), tassel: angle between the axis and lateral branches (TAMALB), tassel: curvature of lateral branches (TCLB), tassel: length of the main axis above the highest lateral branch (TLMAHLB), tassel: number of primary lateral branches (TNPLB). A total of 14 features were analyzed.

### 4.3. Climatic Conditions

The total rainfall in 2017 in Smolice amounted to 36.55 mm and was lower than the multi-annual average sum of precipitation by 12.32 mm. The highest rainfall was recorded in May (54 mm), while the lowest was recorded in June (16 mm). The average air temperature this year was 10.94 °C and was 0.9 °C higher than the multi-annual average temperature. The warmest month in 2017 was August (20 °C), while the lowest temperature was recorded in January (1.2 °C). In 2017, the amount of rainfall and temperature were favorable during the initial development of maize plants. Rainfall was abundant in May, which had a positive effect on maize development.

### 4.4. Genotyping and SilicoDArT and SNP Data Processing

Ninety-four lines were genotyped. Total genomic DNA was extracted from the young leaves of the analyzed forms using the DNeasy Plant Mini Kit (Qiagen GmbH, Hilden, Germany). DNA purity and concentration were determined spectrophotometrically (Thermo Scientific, Waltham, MA, USA). The concentration of all DNA samples was adjusted to 100 ng µL^−1^. The DArTseq analysis was performed by Diversity Arrays Technology Pty Ltd. (Bruce, Australia). The methodology presented below was also used in the research presented by Tomkowiak et al. [38].

DNA samples digestion/ligation reactions were processed according to Kilian et al. [33], but a single PstI-compatible adaptor was replaced with two adaptors, corresponding to PstI- and NspI-compatible sequences, and the assay was performed on the sequencing platform, as described by Sansaloni et al. [12]. The PstI-compatible adapter was designed to include Illumina flowcell attachment sequence, sequencing primer sequence and a “staggered”, varying length barcode region, similar to the sequence reported by Elshire et al. [30]. The reverse adapter contained the flowcell attachment region and NspI-compatible overhang sequence. Only “mixed fragments” (PstI-NspI) were amplified in PCR using the following reaction conditions: Denaturation—1 min at 94 °C, followed by 30 cycles of 94 °C for 20 s, 57 °C for 30 s and 72 °C for 45 s, and the final elongation—72 °C for 7 min. Subsequently, PCR equimolar amounts of amplification products from each sample of the 96-well microtiter plate are bulked and applied to c-Bot (Illumina) bridge PCR, followed by sequencing on Illumina Hiseq2500. Sequencing (single read) reaction included 78 cycles. Sequences generated from each lane were processed using proprietary DArT analytical pipelines. In the primary pipeline, fastq files were first processed to filter out poor-quality sequences, applying more stringent selection criteria to the barcode region compared to the rest of the sequence. In this manner, assigning sequences to specific samples performed in the “barcode split” step was very reliable. Approximately 2,500,000 (+/−7%) sequences per barcode/sample were used in marker calling. Finally, identical sequences were collapsed into “fastqcall files”. These files were used in the secondary pipeline for DArT PL’s proprietary SNP and SilicoDArT (presence/absence of restriction fragments in representation) calling algorithms (DArTsoft14). For association analysis, only DArT sequences meeting the following criteria were selected: One SilicoDArT and SNP within a given sequence (69 bp), minor allele frequency (MAF) >0.25 and missing observation fractions <10%.

### 4.5. Statistical Analysis and Association Mapping

The normality of the distribution of the observed traits was tested using Shapiro–Wilk’s normality test to check whether the analysis of variance (ANOVA) met the assumption that the ANOVA model residuals followed a normal distribution. The homogeneity of variance was tested using Bartlett’s test. Multivariate normality and homogeneity of variance–covariance matrices were tested by Box’s M test. A one-way analysis of variance (ANOVA) was carried out to determine the main effect of lines on the variability of the studied traits. The genetic similarity for each pair of the investigated lines was estimated based on the coefficient proposed by Nei and Li [49]. The lines were grouped hierarchically using the unweighted pair group method of arithmetic means (UPGMA) based on the calculated coefficients. The relationships between the lines were presented in the form of a dendrogram. The relationships between observed traits were assessed based on Pearson’s correlation coefficients and tested with the *t*-test. The results were also analyzed using multivariate methods. Association mapping was performed using a method based on the mixed linear model with the population structure estimated by eigenanalysis (principal component analysis applied to all markers) and modeled using random effects [50,51]. The significance of associations between the traits and SilicoDArT and SNP markers was assessed on the basis of *p*-values corrected for multiple testing using the Benjamini–Hochberg method. Manhattan plots are standard tools used to visualize GWAS results and to identify the genomic regions associated with a given phenotype were used for all 14 maize traits. All analyses were conducted in Genstat 18.2 (VSN International Ltd., Hemel Hempstead, England, UK).

## 5. Conclusions

Breeding companies around the world face many challenges due to the large number of collections of accumulated materials and the need to characterize objects for a wide range of users. Challenges include the necessity of correctly identifying plant materials, analysis of seed material quality, accurate characterization of new plant materials in the collection, distinguishing the core collection, ecotype determination, and genotype and phenotype comparison of the studied plants. All these works aimed to isolate valuable materials and use them for crossbreeding in order to create new cultivars. NGS techniques are applied for innovative genetic identification in plant breeding and, therefore, they are of great importance. The present work demonstrated that this technique has proven to be successful in grouping inbred lines based on their origin. The similarity between the analyzed inbred lines was also calculated, which allowed for the selection of the lines intended for heterotic crosses (e.g., lines 72 and 73, differing in origin and characterized by a high genetic distance at the level of 84%, are a good starting material for crosses). Two markers significantly associated with four analyzed traits were also identified (SNP-4578734 and SilicoDArT-4778900). These markers were subjected to physical mapping, which showed that marker 4578734 was located in the vicinity of the gene encoding anthocyanidin 3-*O*-glucosyltransferase, while marker 4778900 was near the gene encoding starch synthase I. The analyzed maize materials were also phenotyped in this work. The scope of the application of NGS technologies is increasing, as they are constantly being developed. Plant biology has much to gain from better knowledge of plant genomes. Advances in sequencing technology and plant genomic sequencing projects provide a better understanding of developmental and evolutionary processes that form the diversity of life on Earth. As shown by the latest advances in molecular biology, innovative sequencing techniques are the future of plant biology and all fields of life sciences.

## Figures and Tables

**Table 1:** Associations between markers (Silico and SNP) and studied traits found in the genome-wide association mapping with allelic substitution effects (significant associations selected at *p* < 0.05 with correction for multiple testing by the Benjamini–Hochberg method).

Trait	No of Significant Mmarkers			LOD Min			LOD Max			Effect Min			Effect Max			Effect Mean			Total Effect		
	Silico	SNP	Total	Silico	SNP	Total	Silico	SNP	Total	Silico	SNP	Total	Silico	SNP	Total	Silico	SNP	Total	Silico	SNP	Total
ACGC	27	4	31	2.52	2.58	2.52	4.12	3.63	4.12	−0.98	−0.91	−0.98	0.94	0.67	0.94	0.02	−0.12	0.00	0.64	−0.49	0.15
NDSA	52	14	66	2.50	2.55	2.50	3.80	3.74	3.80	−2.85	−2.75	−2.85	3.01	2.54	3.01	1.26	0.01	0.99	65.31	0.12	65.43
NDSSE	62	15	77	2.50	2.56	2.50	5.05	4.24	5.05	−3.04	−2.67	−3.04	3.49	3.03	3.49	0.19	−0.27	0.10	11.85	−4.07	7.77
ACSi	9	1	10	2.58	2.68	2.58	4.11	2.68	4.11	−0.91	−0.57	−0.91	0.67	−0.57	0.67	−0.11	−0.57	−0.16	−1.01	−0.57	−1.58
ACA	14	3	17	2.54	2.54	2.54	3.66	3.27	3.66	−1.02	0.76	−1.02	0.77	0.80	0.80	−0.24	0.78	−0.06	−3.43	2.33	−1.09
TACG	13	9	22	2.53	2.52	2.52	3.25	3.32	3.32	−0.80	−0.86	−0.86	1.06	0.82	1.06	0.35	0.25	0.31	4.56	2.23	6.78
TAMALB	22	5	27	2.54	2.55	2.54	5.28	4.09	5.28	−0.84	0.58	−0.84	0.85	0.87	0.87	−0.38	0.66	−0.19	−8.32	3.29	−5.03
TCLB	28	8	36	2.50	2.51	2.50	3.92	4.10	4.10	−0.62	−0.62	−0.62	0.67	0.46	0.67	0.12	−0.43	0.00	3.29	−3.42	−0.13
TLMAHLB	43	8	51	2.50	2.51	2.50	4.41	3.61	4.41	−0.77	−0.62	−0.77	0.71	0.68	0.71	0.10	−0.12	0.07	4.45	−0.96	3.48
TNPLB	26	3	29	2.51	2.77	2.51	3.55	3.82	3.82	−0.52	0.45	−0.52	0.46	0.46	0.46	−0.04	0.46	0.01	−0.98	1.37	0.39
ACSh	23	8	31	2.52	2.55	2.52	4.00	3.89	4.00	−0.62	−0.62	−0.62	0.67	0.53	0.67	0.26	−0.13	0.16	6.01	−1.07	4.94
ACI	37	17	54	2.52	2.51	2.51	4.51	3.65	4.51	−1.14	−0.71	−1.14	0.94	0.81	0.94	−0.07	0.23	0.02	−2.70	3.91	1.21
PH	7	4	11	2.52	2.52	2.52	3.17	3.61	3.61	−10.92	−11.32	−11.32	15.78	11.35	15.78	5.72	5.22	5.54	40.04	20.90	60.94
HFC	14	5	19	2.51	2.56	2.51	3.63	5.56	5.56	−5.21	−4.73	−5.21	6.59	7.84	7.84	3.46	3.37	3.44	48.49	16.86	65.36
Total	377	104	481																		

**Table 2:** Division of plant material into groups of origin (line numbers from 1 to 94).

Origin Groups of the Lines														
Dent					Flint								Semident	
ID	BSSS	ID/BSSS	Lancaster	ID/Lancaster	F2	F2/EP1	F2/CM7	Flint/BSSS	Flint/ID	Flint/Lancaster	German Flint/F2	Origin Unknown	BSS	Origin Unknown
5,7,8,9, 20,22,23, 33,43,44, 56,58,59, 61,62,64, 65,72,76, 79,80,83, 85,88,89, 90,94.	47, 49, 70, 87.	1,3,4,10, 13,14,17, 19,45,46, 54,57,63, 66,67,75, 77,78,81, 84,86,91, 92.	55	50	74	2,6,15, 18,21, 24,35, 39,41, 51,53.	73	38	40	16, 60, 68.	11, 69, 82, 93.	12, 36, 42, 52.	30, 31, 32, 71.	25, 26, 27, 28, 29, 37, 34, 48.

## Data Availability

The datasets generated during and/or analyzed during the current study are available from the corresponding author on reasonable request.

## Supplementary Materials

The following are available online at https://www.mdpi.com/article/10.3390/ijms22115840/s1, Table S1. Effects of markers (Silico and SNPs) associated with traits found in GWAM with allelic substitution effects (significant associations selected at *p* < 0.05 with correction for multiple testing by the Benjamini-Hochberg method), Table S2. The groups of lines from clustering by unweighted pair group method with arithmetic average (UPGMA) showed in Figure 3.

## Abbreviations

ACA	anthocyanin coloration of anthers,
ACGC	anthocyanin coloration of cob glumes,
ACI	anthocyanin coloration of internodes,
ACSh	anthocyanin coloration of sheath,
ACSi	anthocyanin coloration of silks,
HFC	height of the first cob,
NDSA	number of days from sowing to anthesis,
NDSSE	number of days from sowing to silk emergence,
PH	plant height,
TACG	tassel: anthocyanin coloration at the base of the glume,
TAMALB	tassel: angle between the axis and lateral branches,
TCLB	tassel: curvature of lateral branches,
TLMAHLB	tassel: length of the main axis above the highest lateral branch,
TNPLB	tassel: number of primary lateral branches.
