# Supplementary material for: DArTseq-Based High-Throughput SilicoDArT and SNP Markers Applied for Association Mapping of Genes Related to Maize Morphology
Source: Int J Mol Sci. 2021 May 29;22(11):5840. doi: 10.3390/ijms22115840 (PMC8198497; doi:10.3390/ijms22115840)
Supplement: Supplementary file 1 [file ijms-22-05840-s001.zip › ijms-1219705-supplementary.pdf]

Table S1. Effects of markers (Silico and SNPs) associated with traits found in GWAM with allelic substitution effects (significant associations selected at *p*<0.05 with correction for multiple testing by the Benjamini-Hochberg method).

Marker type	Marker ID	SNP Position REF>ALT*	Frequency of alleles		Trait														
			REF	ALT	ACGC	NDSA	NDSSE	ACSi	ACA	TACG	TAMALB	TCLB	TLMAHLB	TNPLB	ACSh	ACI	PH	HFC
Silico	4778852		0.68	0.32			-2.41											
Silico	5586702		0.59	0.41							-0.68							
Silico	100000720		0.62	0.38														4.45
Silico	100000766		0.66	0.34								-0.62						
Silico	9693684		0.65	0.35								0.52						
Silico	100000820		0.70	0.30								-0.52						
Silico	7050921		0.70	0.30											-0.62			
Silico	2432661		0.71	0.29												-0.67		
Silico	100000914		0.56	0.44	-0.84													
Silico	100000977		0.53	0.47							0.69							
Silico	4581349		0.58	0.42										-0.42				
Silico	100001041		0.53	0.47												0.67		
Silico	100001118		0.62	0.38											0.54			
Silico	4591274		0.63	0.73		-2.02	-2.18											
Silico	2451518		0.67	0.33							-0.76							
Silico	100001217		0.60	0.40										0.40				
Silico	9634523		0.59	0.41											0.57			
Silico	100001252		0.51	0.49										-0.46				
Silico	2493318		0.59	0.41												0.63		
Silico	100001288		0.54	0.46												0.76		
Silico	100001290		0.52	0.48							0.85							

Silico	4774786	0.61	0.39	-0.65												
Silico	100001308	0.61	0.39							-0.76						
Silico	9699175	0.63	0.37										0.64			
Silico	100001321	0.54	0.46							-0.69						
Silico	2384927	0.52	0.48									-0.39				
Silico	100001361	0.58	0.42												11.70	
Silico	9667169	0.66	0.34											0.94		
Silico	2376603	0.58	0.42													4.36
Silico	4586515	0.62	0.38											0.65		
Silico	100001414	0.56	0.44								0.51					
Silico	4766329	0.52	0.48											0.70		
Silico	2431389	0.59	0.41											-0.70		
Silico	4586452	0.68	0.32			1.90										
Silico	100001517	0.67	0.33					0.77								
Silico	100001724	0.56	0.44											0.65		
Silico	4778763	0.60	0.80		1.88											4.20
Silico	100001787	0.63	0.37								0.52					
Silico	9625872	0.52	0.48	0.70												
Silico	100001811	0.62	0.38											0.67		
Silico	100001820	0.62	0.38							-0.72						
Silico	4582403	0.67	0.33					0.70								
Silico	100001857	0.59	0.41						-0.71							
Silico	100001908	0.52	0.96		-2.01	-2.09										
Silico	16725908	0.56	0.44		2.64											
Silico	4584315	0.73	0.27									-0.41				
Silico	4591901	0.67	0.33		2.56											
Silico	4779164	0.64	0.36					-0.74								
Silico	100001948	0.53	0.47				0.67									

Silico	21680386	0.54	0.46									0.37				
Silico	2449731	0.69	0.31									-0.39				
Silico	2515965	0.53	0.47											-0.64		
Silico	2505048	0.70	0.30			2.18										
Silico	4779023	0.56	0.44			-2.07										
Silico	4767754	0.54	0.46			2.76										
Silico	4777576	0.63	0.37										-0.49			
Silico	4591368	0.56	0.44					-0.73								
Silico	4578353	0.62	0.38				-0.74									
Silico	2393694	0.53	0.47											0.68		
Silico	4592941	0.61	0.39	0.84												
Silico	4575695	0.59	0.41								0.61					
Silico	4576551	0.62	0.38									0.41				
Silico	2434873	0.58	0.42							0.54						
Silico	2615548	0.68	0.32	0.66												
Silico	2441153	0.64	0.36							-0.51						
Silico	2572720	0.56	0.44		2.06											
Silico	100002010	0.66	0.69		2.27	2.21										
Silico	2467321	0.62	1.15		2.96	3.27				0.67						
Silico	2562367	0.61	0.77						-0.59					-0.64		
Silico	2453829	0.64	0.36									-0.42				
Silico	4583489	0.66	0.34	-0.78												
Silico	4764905	0.68	0.32												11.74	
Silico	4768977	0.62	0.38													-4.42
Silico	2388390	0.59	0.41		2.10											
Silico	2380229	0.65	0.35										0.67			
Silico	4770467	0.69	0.31	0.69												

Silico	4580276	0.71	0.29			1.99											
Silico	2620819	0.54	0.46											-0.49			
Silico	9687023	0.56	0.44		2.88												
Silico	4588462	0.52	0.48			-2.55											
Silico	4591935	0.60	0.40			2.09											
Silico	4766438	0.70	0.30		2.32												
Silico	4764158	0.65	0.71		2.13	2.18											
Silico	4764438	0.59	0.41	0.65													
Silico	4775686	0.66	0.68		-1.97	-2.28											
Silico	4590553	0.54	0.46	0.71													
Silico	4769249	0.67	0.33		2.53												
Silico	9625741	0.56	0.44	-0.74													
Silico	4769759	0.60	0.40													9.95	
Silico	4775251	0.68	0.32						0.90								
Silico	4583881	0.66	0.34	0.82													
Silico	4586059	0.56	0.88		2.51	2.43											
Silico	16722808	0.57	0.43							0.76							
Silico	4589864	0.64	0.36				0.62										
Silico	4777481	0.54	0.46								-0.53						
Silico	4769755	0.65	0.70			3.32									-1.09		
Silico	4576643	0.70	0.30														4.62
Silico	4777540	0.56	0.44		1.75												
Silico	4593297	0.54	0.46									-0.57					
Silico	4587716	0.60	0.40								0.50						
Silico	4779265	0.69	0.31					0.77									
Silico	4768082	0.54	0.46										-0.49				
Silico	4779340	0.61	0.39									0.68					
Silico	4587505	0.71	0.29	0.84													

Silico	100002084	0.65	0.71		2.67										6.59
Silico	100002093	0.69	0.31									0.68			
Silico	4575949	0.66	0.34		3.01										
Silico	2543171	0.60	1.19		-2.12	-2.71								0.66	
Silico	9646669	0.72	0.28			1.91									
Silico	4776119	0.69	0.31											-0.75	
Silico	2443226	0.52	1.44		2.11	2.48								-0.75	
Silico	4769635	0.59	0.41				-0.60								
Silico	100002149	0.53	0.47									0.60			
Silico	4590886	0.61	0.39								-0.50				
Silico	100002164	0.69	0.31			2.15									
Silico	100002165	0.69	0.62		2.64									-0.94	
Silico	4580038	0.69	0.31						-0.80						
Silico	4764755	0.56	0.44										-0.41		
Silico	2459787	0.55	0.45						0.76						
Silico	9630624	0.64	0.36									0.53			
Silico	4593897	0.53	0.47									0.62			
Silico	21680081	0.62	0.38									0.54			
Silico	16724785	0.51	0.49				0.66								
Silico	4583389	0.56	0.44												4.27
Silico	100002281	0.60	0.80		1.90	2.26									
Silico	100002292	0.53	0.95	-0.98								-0.71			
Silico	16724008	0.65	0.35			2.95									
Silico	100002311	0.60	0.40											-0.66	
Silico	7053929	0.59	0.41					0.70							
Silico	9625870	0.67	0.33							-0.82					

Silico	7050987	0.62	0.38	-0.76									
Silico	7058377	0.72	0.28		2.57								
Silico	4593852	0.66	0.34		2.97								
Silico	4576576	0.68	0.32							0.58			
Silico	100002432	0.68	0.65		2.89							-0.97	
Silico	9624354	0.59	0.41		2.01								
Silico	9679167	0.65	0.35			3.43							
Silico	2495281	0.70	0.30									-1.08	
Silico	5585039	0.69	0.31								0.36		
Silico	5586451	0.56	0.44			2.32							
Silico	9626276	0.55	0.45				-0.94						
Silico	2437315	0.54	0.46									-0.61	
Silico	4774703	0.52	0.48			-2.66							
Silico	9679681	0.67	0.66		-2.00	-1.93							
Silico	4585999	0.54	0.46			-1.96							
Silico	5584399	0.56	0.44					0.86					
Silico	9669478	0.71	0.29										15.78
Silico	9700013	0.63	0.37										12.34
Silico	4582942	0.70	0.30		2.82								
Silico	9682300	0.51	0.49						-0.84				
Silico	9626569	0.53	0.47								-0.38		
Silico	7047697	0.68	0.32		2.55								
Silico	4768272	0.69	0.31							0.53			
Silico	2479835	0.59	0.41									-0.85	
Silico	9690137	0.56	0.88		2.48	2.81							
Silico	9694682	0.60	0.40			3.11							

Silico	2475782	0.60	0.40		2.58									
Silico	4779485	0.70	0.30	0.69										
Silico	100002669	0.58	0.42					-0.62						
Silico	9625310	0.69	0.31										-0.66	
Silico	4576193	0.70	0.30										-1.14	
Silico	4586570	0.68	0.32										-0.95	
Silico	9629319	0.66	0.34		2.49									
Silico	4588216	0.64	0.36			2.78								
Silico	4591640	0.67	0.33					-0.65						
Silico	100002757	0.65	0.35		2.73									
Silico	16721565	0.65	0.35			3.49								
Silico	4591589	0.71	0.29		2.53									
Silico	9625942	0.67	0.33				-1.02							
Silico	9691928	0.60	0.40										0.65	
Silico	21697334	0.73	0.27						0.55					
Silico	4584261	0.58	0.42			2.79								
Silico	21692047	0.65	0.70		2.89	3.17								
Silico	9631317	0.58	0.42								-0.52			
Silico	4766483	0.70	0.30							0.71				
Silico	4575773	0.68	0.32											5.31
Silico	2532528	0.58	0.42							-0.60				
Silico	100002947	0.68	0.32		2.65									
Silico	9670753	0.68	0.32			2.56								
Silico	2537186	0.57	0.43								-0.40			
Silico	16723202	0.54	0.46									0.51		
Silico	9681025	0.70	0.30		2.61									
Silico	9626799	0.56	0.44				-0.76							

Silico	9669360	0.70	0.30		2.27												
Silico	100003041	0.68	0.32				-0.91										
Silico	4577187	0.60	0.80		1.83	1.85											
Silico	100003095	0.69	0.31										0.39				
Silico	4576676	0.59	0.41												-0.68		
Silico	4586831	0.53	0.47									-0.51					
Silico	2497886	0.62	0.38													-10.92	
Silico	9690376	0.64	0.36								-0.55						
Silico	7054381	0.69	0.31	0.87													
Silico	4779610	0.51	0.49										0.41				
Silico	100003857	0.68	0.32									0.67					
Silico	100000158	0.68	0.32														4.50
Silico	2425780	0.52	0.48										-0.37				
Silico	4584415	0.55	0.89											0.53			-5.21
Silico	5585990	0.69	0.31					-0.72									
Silico	2411288	0.67	0.33	-0.69													
Silico	2428853	0.60	0.40						0.75								
Silico	5586833	0.69	0.62		-2.85	-2.99											
Silico	2442751	0.53	0.47								-0.45						
Silico	2533101	0.55	0.45										0.43				
Silico	100000194	0.60	0.81			-2.15									0.89		
Silico	7047334	0.59	0.41	-0.68													
Silico	100000198	0.65	0.35							-0.77							
Silico	2505163	0.58	0.42				-0.63										
Silico	2445912	0.54	0.46									0.57					
Silico	5583194	0.55	0.45					-0.63									
Silico	2445784	0.63	0.37									-0.62					

Silico	2453002		0.53	0.47				-0.65							
Silico	2493742		0.67	0.33									0.49		
Silico	4768984		0.61	0.39			-2.11								
Silico	100011275		0.69	0.31					0.76						
Silico	100011301		0.52	0.48								-0.55			
Silico	4775974		0.65	0.35								0.53			
Silico	100011351		0.69	0.62		1.90	2.54								
Silico	100011425		0.70	0.30								-0.52			
Silico	100011506		0.55	0.45									0.57		
Silico	2412628		0.58	0.42						-0.64					
Silico	4778393		0.54	0.46	-0.84										
Silico	4771578		0.63	0.74		1.99	1.97								
Silico	21679923		0.68	0.32								0.53			
SNP	2583789	???	0.71	0.29					0.76						
SNP	4779712		0.60	0.40			2.37								
SNP	4771733	51: C>A	0.51	0.49							-0.51				
SNP	9708055		0.63	0.37								-0.59			
SNP	5590247		0.61	0.39										-0.65	
SNP	4771683		0.56	0.44											4.51
SNP	4769530		0.65	0.35									-0.49		
Silico	2429464		0.59	0.41		1.91									
SNP	9682134		0.59	0.41										0.81	
SNP	9687721		0.58	0.42										-0.69	
Silico	4581381		0.68	0.32						-0.65					
SNP	2553774		0.63	0.37							-0.59				
SNP	4773377		0.60	0.40		2.03									
SNP	2605799		0.54	0.46											4.81

Silico	9629607	0.51	0.49		-2.17									
SNP	7060210	0.63	0.37											-4.73
Silico	4765147	0.57	0.43								0.45			
SNP	2393909	0.56	0.44						0.49					
SNP	4586030	0.65	0.35									0.77		
SNP	2458887	0.72	0.28				0.87							
SNP	4772403	0.51	0.49									-0.64		
SNP	2379065	0.67	0.33								-0.48			
SNP	2511088	0.55	0.45		2.27									
SNP	2588741	0.67	0.33									0.78		
SNP	4770911	0.51	0.49							0.45				
SNP	4771928	0.54	0.46							0.46				
SNP	9699008	0.68	0.32								-0.62			
SNP	2552647	0.55	0.45	2.08										
SNP	2464547	0.53	0.94			0.78						0.71		
SNP	4579818	0.63	1.11	-1.93	-1.86								-11.32	
SNP	2458775	0.68	0.32					-0.57						
SNP	4776124	0.58	0.42					0.46						
SNP	9709009	0.70	0.30			0.79								
SNP	2568795	0.66	0.34		-2.26									
SNP	2430868	0.51	0.99								0.46	0.69		
SNP	4770960	0.54	0.46		-1.82									
SNP	4776767	0.52	0.48				0.59							
SNP	4765115	0.53	0.47	2.35										
SNP	2523575	0.53	0.47									0.72		
SNP	4579446	0.68	0.32		-1.92									
SNP	4767346	0.53	0.47				0.61							

SNP	4581844	0.65	0.35										-0.71		
SNP	4582717	0.68	0.32										0.74		
SNP	4575760	0.52	0.48		-2.05										
SNP	7049087	0.72	0.28		-2.45										
SNP	4588629	0.53	1.42		2.05								-0.71	9.90	
SNP	4578734	0.67	1.33	-0.91	-2.25	-2.46							0.75		
SNP	4577915	0.57	0.43												4.43
SNP	4576114	0.55	0.89							-0.50			0.61		
SNP	7049478	0.60	0.40							-0.53					
SNP	4576906	0.53	0.47				0.68								
SNP	4576836	0.57	0.43		-1.72										
SNP	4592788	0.57	0.86			-1.84						0.51			
SNP	4582907	0.52	0.48					0.64							
SNP	4584915	0.65	0.35									0.53			
SNP	4767698	0.60	0.40									-0.46			
SNP	4770352	0.70	0.30				0.82								
SNP	4581826	0.60	0.40						-0.49						
SNP	7059865	0.54	0.92		2.54	3.03									
SNP	9701186	0.56	0.44						-0.61						
Silico	4775850	0.66	0.34								0.41				
SNP	16725691	0.51	0.49							0.62					
SNP	4778107	0.57	0.43		-2.03										
SNP	4770814	0.58	0.84		2.38	2.68									
SNP	4776723	0.53	0.47				-0.66								
SNP	4583764	0.63	0.37						-0.48						
SNP	2445150	0.59	0.41											10.97	
SNP	4576866	0.60	0.40			2.67									

SNP	7060833	0.68	0.32			-2.67											
Silico	4765267	0.60	0.40								0.50						
SNP	4589214	0.51	0.99													11.35	7.84
SNP	4590030	0.54	0.46				-0.57										
SNP	4583980	0.52	0.48			-2.04											
SNP	2518079	0.68	0.32								-0.62						
SNP	4777619	0.53	0.47		1.88												
Silico	2390931	0.66	0.34									0.54					
SNP	7060243	0.63	0.37	0.62													
Silici	4766343	0.66	0.34			2.02											
SNP	4586601	0.53	0.47									0.68					
Silico	4778895	0.52	0.48										0.37				
SNP	2479238	0.68	0.32											-0.52			
Silico	100000332	0.55	0.45			-2.23											
SNP	4593498	0.56	0.44	0.67													
SNP	7060505	0.71	0.29		-2.75												
SNP	4575988	0.60	0.40									-0.62					
SNP	4773530	0.61	0.39									-0.51					
Silico	4775531	0.54	0.46									-0.55					
SNP	9707399	0.64	0.36					0.80									
SNP	7059799	0.59	0.41						0.70								
SNP	4586817	0.53	0.47			1.98											
Silico	16725112	0.56	0.44					-0.74									
SNP	4589328	0.68	0.63			-2.19									0.76		
Silico	4592102	0.72	0.28	-0.76													
SNP	4772738	0.55	0.45	-0.87													
SNP	7061374	0.56	0.44							0.58							
SNP	4577625	0.55	0.89						-0.86						- 0.70		

SNP	4577625	0.59	0.41					-0.79						
SNP	4772896	0.68	0.32				0.77							
Silico	9666967	0.60	0.40	-0.75										
SNP	9701846	0.65	0.35					0.77						
Silico	100000368	0.55	0.45								-0.52			
Silico	4768443	0.51	0.98		1.80	1.77								
SNP	4764785	0.65	0.35											0.68
SNP	7050483	0.66	0.34									0.46		
Silico	4778264	0.57	0.43									0.36		
Silico	7051609	0.54	0.46				-0.71							
Silico	4576233	0.59	0.41							0.50				
Silico	2508332	0.66	0.34										0.60	
Silico	4588066	0.58	0.84			-2.21								0.72
Silico	9669855	0.70	0.30								-0.71			
Silico	4779929	0.70	0.30						-0.67					
Silico	4586667	0.55	0.45			-1.98								
Silico	2515902	0.54	0.46										0.57	
Silico	7051351	0.68	0.32									-0.37		
Silico	9624307	0.56	0.44								0.58			
Silico	4591566	0.60	0.40									-0.36		
Silico	2592246	0.61	0.39								-0.77			
Silico	9681167	0.58	0.42											-0.72
Silico	4587371	0.54	0.46						-0.62					
Silico	2435892	0.52	0.97		1.90	2.02								
Silico	7048006	0.54	0.46										0.47	
Silico	4774664	0.60	0.40			-2.04								
Silico	4591904	0.58	0.85								-0.52			0.65
Silico	4585663	0.58	0.42								-0.61			

Silico	21697548	0.56	0.44	0.75													
Silico	100000456	0.53	0.47												0.61		
Silico	7051647	0.62	0.38								-0.57						
Silico	2393750	0.56	0.44						0.73								
Silico	4578921	0.60	0.40				0.57										
Silico	4588775	0.54	0.91			-2.07									0.70		
Silico	4765165	0.63	0.73									-0.63			0.65		
Silico	7060522	0.66	0.34			-2.35											
Silico	4589697	0.72	0.28								0.54						
Silico	2604248	0.52	0.48												-0.69		
Silico	4776242	0.69	0.31								0.49						
Silico	4576343	0.51	0.49									0.52					
Silico	4776575	0.52	0.48									0.50					
Silico	7048525	0.56	0.88			-1.89									0.62		
Silico	100000048	0.57	0.43											0.50			
Silico	9696462	0.65	0.35		-2.28												
Silico	4585394	0.58	0.42					-0.88									
Silico	4579853	0.58	0.42	0.94													
Silico	4778900	0.63	1.48		-1.88	-1.90				0.62						-10.55	
Silico	9674255	0.57	0.86			1.85						0.47					
Silico	100000501	0.68	0.65			-2.05							0.46				
Silico	4765221	0.59	0.41	-0.67													
Silico	4766795	0.56	0.44								0.59						
Silico	4587481	0.65	0.35														4.82
Silico	4590009	0.51	0.49		-2.21												
Silico	4587096	0.54	0.46								0.64						
Silico	4582535	0.70	0.30								-0.57						

Silico	4580542	0.72	0.28					0.50				
Silico	7053852	0.60	0.40									4.58
Silico	4584283	0.63	0.37		-2.50							
Silico	9628015	0.63	0.37						0.64			
Silico	9665124	0.52	0.48						0.55			
Silico	7053916	0.56	0.44		-1.92							
Silico	7050893	0.51	0.49				0.79					
Silico	4769261	0.67	0.33	0.84								
Silico	16720795	0.57	0.43		-2.21							
Silico	4776507	0.54	0.46						0.55			
Silico	4767650	0.59	0.41			-1.94						
Silico	4585220	0.54	0.46				0.81					
Silico	7058553	0.68	0.32									5.56
Silico	100000543	0.60	0.40							0.42		
Silico	4587749	0.68	0.32						-0.62			
Silico	4585833	0.52	0.48						0.56			
Silico	4774305	0.63	0.37					0.54				
Silico	4765871	0.61	0.39								-0.49	
Silico	4576824	0.51	0.49	-0.83								
Silico	4577604	0.70	0.30			-3.04						
Silico	4593337	0.57	0.43			-2.10						
Silico	7054077	0.67	0.33					0.50				
Silico	4592058	0.56	0.44			-2.08						
Silico	7047961	0.55	0.90		-2.03	-2.64						
Silico	4579437	0.66	0.68			2.14					-0.60	
Silico	4585534	0.68	0.32					-0.52				
Silico	4585588	0.58	0.42	0.61								
Silico	4579719	0.66	0.34				0.90					

Silico	4583924	0.51	0.49			-2.10											
Silico	7047169	0.65	0.35											0.56			
Silico	4766434	0.64	0.36								-0.45						
Silico	4764784	0.66	0.34						-0.78								
Silico	4576771	0.61	0.39								0.48						
Silico	4774853	0.55	0.45									-0.74					
Silico	4588492	0.68	0.32						1.06								
Silico	9627504	0.70	0.30														4.86
Silico	9638854	0.58	0.42									-0.68					
Silico	4774828	0.53	0.47			-1.93											
Silico	4585343	0.63	0.37											0.55			
Silico	21698627	0.50	0.50											-0.59			
Silico	4576226	0.58	0.42											0.57			
Silico	4776458	0.70	0.30											0.51			
Silico	4766629	0.52	0.48								0.47						
Silico	4589770	0.51	0.49							0.68							
Silico	4589146	0.72	0.28							-0.78							
Silico	4779031	0.68	0.32									0.56					
Silico	4767556	0.58	0.42							-0.66							
Silico	100000608	0.68	0.32								0.54						
	Total			31	66	77	10	17	22	27	36	51	29	31	54	11	19

Table S2. The groups of lines from clustering by unweighted pair group method with arithmetic average (UPGMA) showed in Figure 3.

Group number	Lines number
Group 1	2, 6, 9, 15, 16, 17, 18, 19, 21, 22, 25, 26, 27, 28, 29, 30, 31, 32, 35, 38, 39, 40, 41, 42, 51, 53, 60, 68, 69, 71, 74, 82, 93
Group 2	80
Group 3	3, 11, 36, 37, 52, 54, 56, 57, 58, 61, 65, 66, 67, 75, 79, 86, 87, 88, 91
Group 4	1, 4, 5, 7, 8, 10, 12, 13, 14, 20, 23, 24, 33, 34, 43, 44, 45, 46, 47, 48, 49, 50, 55, 59, 62, 63, 64, 70, 76, 77, 78, 81, 83, 84, 85, 89, 90, 92, 94
Group 5	72, 73
